# Dermoscopic characteristics of melanoma according to the criteria „ulceration“ and „mitotic rate“ of the AJCC 2009 staging system for melanoma

**DOI:** 10.1371/journal.pone.0174871

**Published:** 2017-04-11

**Authors:** Teresa Deinlein, Edith Arzberger, Iris Zalaudek, Cesare Massone, Juan Garcias-Ladaria, André Oliveira, Günter Schulter, Rainer Hofmann-Wellenhof

**Affiliations:** 1 Department of Dermatology, Medical University of Graz, Graz, Austria; 2 Department of Dermatology, Galliera Hospital, Genoa, Italy; 3 Hospital General Universitari de Valencia, Valencia, Spain; 4 Department of Dermatology, Hospital de Curry Cabral—Centro Hospitalar de Lisboa Central, Lisboa, Portugal; 5 Department of Psychology, Biological Psychology Unit, Karl-Franzens-University Graz, Graz, Austria; Centro Cardiologico Monzino, ITALY

## Abstract

**Objective:**

The present study was conducted to identify possible dermoscopic patterns, associated with mitotic rate > 1/mm^2^, histological ulceration in melanoma and metastatic disease.

**Methods:**

For this retrospective data analysis all clinical and dermoscopic digital images of primary malignant melanomas between 2008 and 2013 documented at the Department of Dermatology Graz were included, using the internal image data-base. 550 patients with 559 melanomas were included.

**Results:**

While clinical or dermoscopic analysis considered ulceration to be present in 120 (21.5%) and 117 (20.9%) of all lesions, respectively, histopathology reported ulceration in only 96 cases (17.2%). The presence of milky-red areas, shiny-white streaks, a blue-white veil and blue-grey areas in dermoscopy is highly correlated with histological ulceration and a mitotic rate > 1/mm^2^. The dermoscopic patterns shiny-white streaks, milky-red areas and blue-white veil were also significantly associated with development of distant metastases.

**Conclusion:**

Our study proves a significant correlation between the dermoscopic patterns "blue white veil“, "milky-red areas”and "shiny-white streaks”and the histological findings "ulceration”and "mitotic rate > 1/mm^2^“. Furthermore these dermoscopic patterns are highly related to distant metastases. Thus, dermoscopy renders earlier prognostic statements possible.

## Introduction

Dermoscopy is a non-invasive diagnostic tool allowing a better visualisation of morphological details of pigmented skin lesions. This technique bridges the gap between the macroscopic clinical dermatology and the microscopic dermatopathology [1,2].

Numerous studies proved dermoscopy to be more accurate than naked eye examination for the diagnosis of cutaneous melanoma. Furthermore, the addition of dermoscopy can reduce the rate of excision for diagnostic verification significantly [3–5].

2009 the final AJCC version for staging and classification of melanoma was released.

An important modification was that ulceration histologically, besides tumor thickness, is regarded as a relevant prognostic factor. Moreover, ulceration leads to classification into B of stage I, II and III tumors [1].

In addition, the mitotic rate was worked out as an important and independent criterion concerning the survival rate of stage I patients. A strong correlation has been found between increasing mitotic rate and decreasing survival.

Therefore, a mitotic rate ≥1/mm^2^ together with tumor thickness and histological ulceration represents the most important factor for stage I melanomas [1].

Accordingly, the aim of this paper was to evaluate, whether there are any dermoscopic structures correlating with a mitotic rate ≥1/mm^2^ or an ulceration histologically. Moreover, we examined, to what extent a clincal or dermoscopic ulceration coexists with a histological ulceration.

## Methods

For this current study all clinical and dermoscopic digital images of patients with primary malignant melanoma documented at the Department of Dermatology Graz between 2008 and 2013 were included, using the internal image data-base.

The last medical and histological reports of each patient were saved in an individual folder, containing the respective clinical and dermoscopic pictures of the patients´ lesions.

Additionally an Excel-file was created, containing several clinical and histological parameters.

The evaluation process of the pictures was done by two experienced dermatologists (EA and RHW). In case of disagreement a third dermatologist (IZ) was consulted. Approximately 5% of the cases were discussed with the third dermatologist, but the exact intra- and inter-observer correlations were not calculated.

We evaluated the lesions applying the dermoscopic patterns proposed by the international consensus meeting [6] and added the criterion "shiny-white streaks" indicating malignant melanoma (Table 1). For lesions located in the face or on the palms and soles the following criteria for evaluation were applied additionally:

- face: pseudo-network, asymmetric pigmented follicles, anular-granular structures
- palms and soles: parallel-furrow pattern, lattice-like pattern, fibrillar pattern, parallel-ridge pattern

**Table 1:** Frequency of dermoscopic patterns related to histological ulceration, mitotic rate, sentinel lymph node and distant metastases.

Dermoscopic pattern	Total n = 559	Ulcerationn = 96	No ulcerationn = 463	Mitotic rate≥ 1/mm^2^n = 121	Mitotic rate < 1/mm^2^n = 438	Positive Sentinelnoden = 45	Negative Sentinelnode/ not performed n = 514	Distant metastases n = 29	No distant metastasesn = 530
**Network**	**245(43.83%)**	**26**	**219**	**45**	**200**	**38**	**207**	**7**	**238**
Regular	101	9	92	9	92	5	96	2	99
Irregular	144	17	127	36	108	33	111	5	139
**Negative pigmentnetwork**	**22(3.94%)**	**4**	**18**	**4**	**18**	**3**	**19**	**0**	**22**
**Globules**	**268(47.94%)**	**26**	**242**	**63**	**205**	**21**	**247**	**11**	**257**
Regular	2	0	2	1	1	0	2	0	2
Irregular	259	26	233	55	204	20	239	10	249
Peripheral	7	0	7	7	0	1	6	1	6
**Streaks**	**150(26.83%)**	**10**	**140**	**33**	**117**	**12**	**138**	**4**	**146**
Regular	0	0	0	0	0	0	0	0	0
Irregular	119	4	115	23	96	10	109	3	116
Peripheral	31	6	25	10	21	2	29	1	30
**Shiny-white streaks**	**124(22.18%)**	**31**	**93**	**50**	**74**	**23**	**101**	**16**	**108**
**Blue-withish veil**	**202(36.16%)**	**38**	**164**	**76**	**126**	**24**	**178**	**21**	**181**
**Milky-red areas**	**121(21.65%)**	**39**	**82**	**48**	**73**	**24**	**97**	**14**	**107**
**Blotches**	**207(37.03%)**	**31**	**176**	**52**	**155**	**17**	**190**	**12**	**195**
Regular	3	0	3	1	2	0	3	0	3
Irregular	203	30	173	50	153	17	186	12	191
Peripheral	1	1	0	1	0	0	1	0	1

For statistical analysis of the data frequency comparisons with the Chi-Quadrat-Test were applied. For doing this we used the SPSS-23 software.

## Ethical aspects

As we used patients´ data, an ethics committee approval was obtained before starting the data collection.

## Results

A total of 550 patients with 559 melanomas were included in our retrospective study (272 women; 49.5% and 278 men; 50.5%). The average age of patients was 64.5 years at time of diagnosis.

97 were melanoma in situ, 251 pT1a, 67 pT1b, 35 T2a, 14 T2b, 31 T3a, 25 T3b, 11 T4a, 28 T4b. 190 melanomas were located in the face, 207 at the trunk, 122 at the extremities and 40 at the palms and soles.

While clinical or dermoscopic analysis considered ulceration to be present in 120 (21.5%) and 117 (20.9%) of all lesions, respectively, histopatholgy reported ulceration in only 96 (17.2%).

In 121 (21.6%) a "mitotic rate ≥1/mm^2^" was reported in histopathology and 96 melanomas (17.2%) showed ulceration histologically. It became apparent that the dermoscopic patterns "shiny-white streaks“, "milky-red areas”and a "blue-whitish veil”were highly correlated with both histological findings "ulceration”and "mitotic rate > 1/mm^2^", respectively (p < 0,001).

318 (56.9%) invasive melanomas had a tumor thickness less than 1mm. 43 (14.4%) of these had a mitotic rate ≥1/mm^2^. The dermoscopic features "peripheral streaks”, "shiny-white streaks", a "blue-whitish veil" and "blotches" were highly correlated with a mitotic rate ≥1/mm^2^ in these tumors, respectively.

In 110 patients a sentinel-lymphnode biopsy was performed and 45 (40.9%) showed a positive result. No association could be substantiated concerning dermoscopic patterns and a positive sentinel-lymphnode.

29 (5.2%) of the patients suffered from distant metastases over the course of time. The median follow-up time was 22 months. The analysis of dermoscopic patterns proved that "shiny-white streaks“, "milky-red areas”as well as a "blue-whitish veil”were suggestive of the presence of distant metastases (p< 0,001) (Table 1).

At time of primary diagnosis the frequency of stages according to the AJCC 2009 classification was divided as follows: IA 1 patient, IB 2 patients, IIA 2 patients, IIB 1 patient, IIC 5 patients, IIIA 3 patients, stage IIIB 7 patients and stage IV 8 patients. Table 1 shows the distribution of the dermoscopic patterns "milky-red areas", "shiny-white streaks" and "blue-whitish veil" depending on stage. Significant stage specific conclusions cannot be made (Table 2).

**Table 2:** Distribution of the dermoscopic patterns "milky-red areas", "shiny-white streaks" and "blue-whitish veil" in primary melanomas leading to metastatic disease depending on stage.

Stage (according to the AJCC 2009 classification)	n	milky-red areas	shiny-white streaks	blue-withish veil
**IA**	1	0	0	1
**IB**	2	1	2	2
**IIA**	2	0	1	2
**IIB**	1	1	0	1
**IIC**	5	3	2	5
**IIIA**	3	2	3	3
**IIIB**	7	5	3	3
**IIIC**	0	0	0	0
**IV**	8	2	5	4

## Discussion

A good correlation between some dermoscopic features and invasive melanomas has already been confirmed [2,7–12]. Several authors [2,7–11] proved the presence of radial streaming, atypical vascular pattern and grey blue areas to be associated with melanomas thicker than 0.75mm. However, there is little known about the correlation between dermoscopic findings in melanomas and both histological ulceration and mitotic rate ≥1/mm^2^ or the prognosis of the disease [13,18].

**Fig 1:**
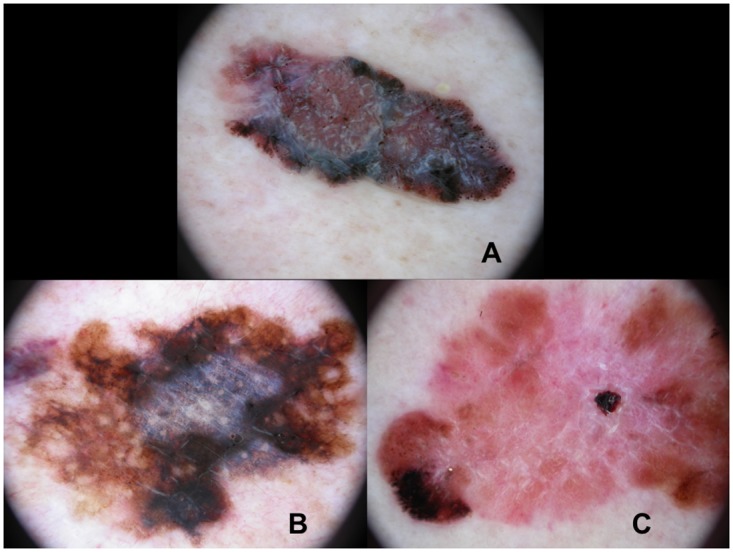
Histologically ulcerated melanomas showing shiny-white streaks (A), a blue-whitish veil (B) and milky-red areas (C).

**Fig 2:**
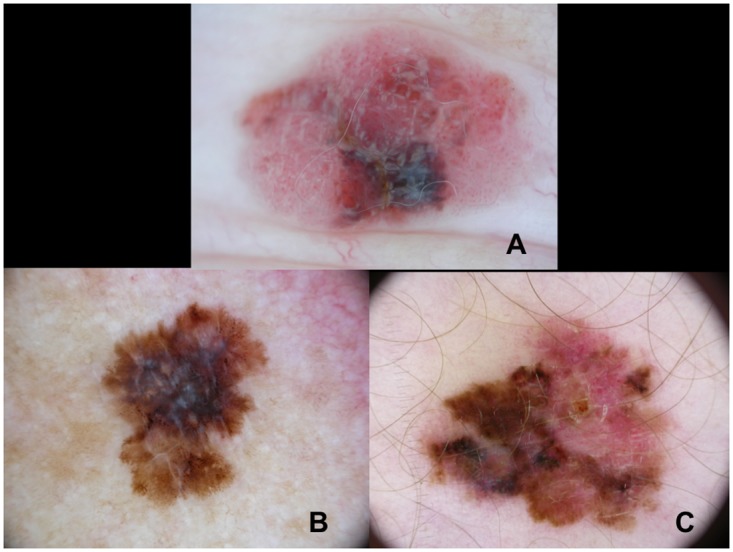
Melanomas with a mitotic rate >1/mm^2^ revealing shiny-white streaks and milky-red areas (A), a blue-whitish veil (B) and milky-red areas (C). These tumors had a tumor thickness less than 1mm.

In our study, the presence of "shiny-white streaks", "milky-red areas" and a "blue-whitish veil" in dermoscopy was highly associated with an ulceration in histology and a mitotic rate ≥1/mm^2^ (Figs 1 and 2). These results are of considerable importance, since a histological ulceration and a mitotic rate ≥1/mm^2^ had been worked out as independent prognostic factors in cutaneous melanoma [1]. Moreover, some studies suggest a high mitotic rate to be a more important prognostic factor than histological ulceration and tumor thickness [14,15].

Underlining the prognostic importance of a high mitotic rate, the work of Shen et al. [16] proved that melanomas with a very high mitotic activity (>10/mm^2^) were predominantly thick and ulcerated nodular tumor subtypes. Furthermore, the recent study of Wang et al. [17] proved a strong correlation between mitotic rate and Ki-67 expression. These results emphasize again the prognostic relevance of a high mitotic rate, since the proliferative activity, as defined by Ki-67 monoclonal antibody, has already been proven to be an unfavourable prognostic factor in melanomas [18].

96 (17.2%) of our melanomas showed ulceration in histology. Strikingly, we found a poor correlation between clinical or dermoscopic and histological ulceration. We speculate that this finding might be due to trauma or a previous punch biopsy.

As ulceration is an important criterion in the AJCC 2009 classification system, the question of whether this might be a result of under-reporting in histopathology or over-estimation in a clinical setting requires further study.

**Fig 3:**
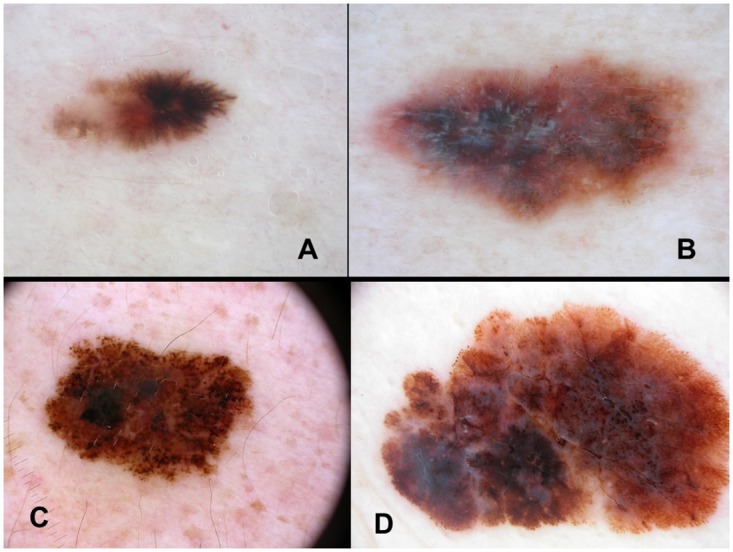
Melanomas with a tumor thickness of less than 1mm and a mitotic rate ≥1/mm^2^ showing peripheral streaks (A), shiny-white streaks (B), irregular blotches (C) and a blue-whitish veil (D).

Concerning invasive melanomas with a tumor thickness of less than 1mm, our study shows a strong association between the dermoscopic patterns "peripheral streaks", "shiny-white streaks", "blotches" and a "blue-whitish veil" and a mitotic rate ≥1/mm^2^ in these tumors (Fig 3). These results indicate that even in thin melanomas those above mentioned unfavourable criteria are valid suggesting a high proliferative activity in melanomas.

The work of González-Álvarez et al. [13] revealed the presence of blotches and ulceration dermoscopically and the absence of a pigmentnetwork, as well as an ulceration in histopathology to be highly correlated with a positive sentinel-lymphnode biopsy. In this present study, however, this correlation could not be substantiated. Our results are in accordance with one retrospective study [19] revealing no dermoscopic features associated with a positive sentinel-lymphnode.

**Fig 4:**
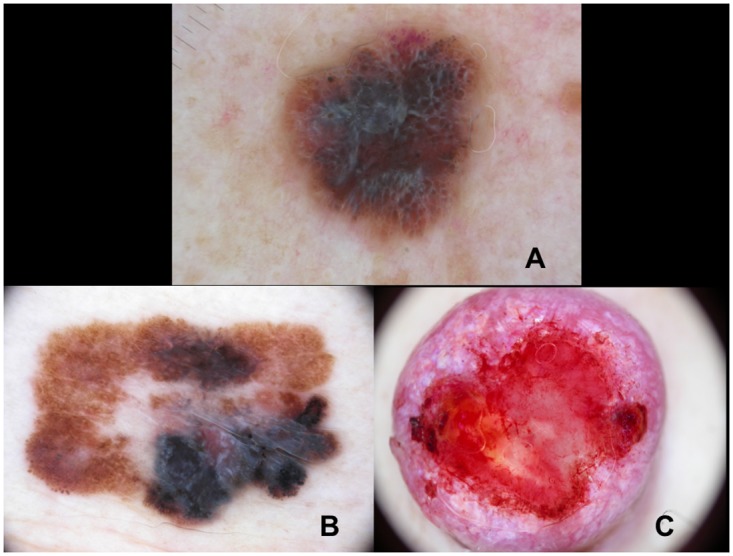
Melanomas exhibiting distant metastases show shiny-white streaks (A), a blue-whitish veil (B) and milky-red areas (C).

29 (5.2%) of our patients developed distant metastases over the course of time. Our results confirm the presence of the dermoscopic patterns "shiny-white streaks", "milky-red areas" and a "blue-whitish veil" to be highly associated with distant metastases (Fig 4). These results therefore allow a precautious prognostic estimation. Limitations of this study are the retrospective design and the fact that intra- and inter-observer correlations for the different clinical and dermoscopic criteria were not evaluated.

In conclusion, our results prove a strongly significant correlation between the dermoscopic patterns "shiny-white streaks", "milky-red areas" as well as a "blue-whitish veil" and the unfavourable histological findings "ulceration" and "mitotic rate > 1/mm^2^".

Furthermore, we verified an association between these patterns and the presence of distant metastases.

Focussing on thin melanomas of less than 1mm thickness, it has to be emphasised that the above mentioned dermoscopic features are also of relevance. Tumors exhibting these patterns are consequently classified as stage Ib. Hence, even in thin melanomas, dermoscopy proves to be a method of high accuracy. The correlation to histology is striking, thus underlining the significance of dermoscopy for prognosis even in early tumor stages.

## Supporting information

S1 File Data base melanoma. (XLSX)
